# Molecular Epidemiology of Methicillin-Resistant *Staphylococcus aureus* in Horses, Cats, and Dogs Over a 5-Year Period in France

**DOI:** 10.3389/fmicb.2017.02493

**Published:** 2017-12-13

**Authors:** Marisa Haenni, Pierre Châtre, Céline Dupieux-Chabert, Véronique Métayer, Michèle Bes, Jean-Yves Madec, Frédéric Laurent

**Affiliations:** ^1^Unité Antibiorésistance et Virulence Bactériennes, French Agency for Food, Environmental and Occupational Health Safety, Claude Bernard University Lyon 1, Lyon, France; ^2^Bacteriology Department, Institut for Infectious Agents, French National Reference Centre for Staphylococci, Hospices Civils de Lyon, Lyon, France; ^3^INSERM U1111, CNRS, International Center for Infectiology Research, UMR5308, École Normale Supérieure de Lyon, Claude Bernard University Lyon 1, Lyon, France; ^4^Institut for Pharmaceutical and Biological Sciences of Lyon, Lyon, France

**Keywords:** MRSA, *mecA*, clone, horses, cats, dogs, veterinary microbiology

## Abstract

Methicillin-resistant *Staphylococcus aureus* (MRSA) has been reported as a worldwide pathogen in humans and animals including companion animals, i.e., cats, dogs, and horses. France lacked a comprehensive nationwide study describing the molecular features of MRSA circulating among companion animals over a large period of time. Here is reported the characterization of 130 non-duplicate clinical MRSA isolates collected from those three animal species from 2010 to 2015 through the French national Resapath network. Characterization of isolates was performed using phenotypic (antimicrobial susceptibility tests) and molecular (DNA arrays, *spa*-typing) methods. A horse-specific epidemiology was observed in France with the large dissemination of a unique clone, the CC398 clone harboring a Staphylococcal chromosomal cassette *mec* (SCC*mec*) type IV and *spa*-type t011. It was even the unique clone collected in 2015 whereas the clone CC8 USA500 (SCC*mec* type IV), classically described in horses, was present until 2014. Contrarily, cats and dogs were mainly infected by human-related MRSA isolates, i.e., clones usually reported in human infections, thus mirroring the human epidemiology in hospitals in France. Isolates belonging to the CC398 clone (SCC*mec* type IV or V) were also identified in 21.4% of dogs’ and 26.5% of cats’ MRSA isolates. In order to differentiate human-related from CC398 MRSA, tetracycline-resistance [or *tet*(M) detection] could be useful since this resistance is scarce in human-related strains but constant in CC398 MRSA isolates. In all, our data give a nationwide epidemiological picture of MRSA in companion animals over a 5-year period in France, adding further epidemiological information on the contribution of those animal species to a major public health issue. Considering the wide dissemination of CC398 MRSA isolates and the fact that 11/64 (17.2%) of them presented the Immune Evasion Cluster which enhances CC398 capacities to colonize humans, a specific attention should be paid in the coming years to determine the risk associated to the transmission in people in frequent contacts with companion animals. Our data also show that the prevalence of MRSA has likely decreased in cats, dogs, and horses between 2012 and 2015 in France. This trend should be monitored in the years to come.

## Introduction

The role of animals in the transmission of methicillin-resistant *Staphylococcus aureus* (MRSA), has been extensively documented in the case of the livestock-associated MRSA (LA-MRSA) complex clonal (CC)398 in pigs and people in contact (Aires-de-Sousa, 2017). Whereas pig farming is restricted to specific areas and human communities, companion animals (including cats, dogs, and horses) are geographically wide spread and mostly not related to occupational activities. It has been estimated that 19 millions of cats and dogs live in households in France and that 750,000 equines (horses and ponies) are owned by stud farms or families for horse riding. Deciphering the routes of transmission of MRSA in companion animals is an ongoing issue. Even though all three animal species are in frequent and close contacts with humans, the epidemiology of MRSA is quite divergent, with cats and dogs being frequently colonized or infected by human clones and horses showing a species-specific MRSA distribution.

Several studies showed that MRSA clones circulating in cats and dogs are similar to the ones identified in humans and belong mostly to hospital-acquired clones (HA-MRSA) (Loeffler and Lloyd, 2010). This is clinically important since HA-MRSA isolates usually carry more virulence genes [such as those coding for enterotoxins or the toxic shock syndrome toxin (TSST)], and also more resistance genes than MRSA originating from animals (Mutters et al., 2016; Ballhausen et al., 2017). The only exception is the *tet*(M) gene, which is highly prevalent in LA-MRSA and MRSA isolates of animal origin but rare in HA-MRSA. This human-related epidemiology suggests that humans may be the source of MRSA isolated in cats and dogs, although those animals may act as a secondary reservoir capable of human re-infections in specific contexts (Harrison et al., 2014; Bierowiec et al., 2016). Reports on MRSA in cats and dogs from England, Germany, Switzerland, and Portugal described the predominance of the sequence type (ST)22 clone, also known as EMRSA-15 or Barnim clone, which is prevalent in human patients in the same countries (Strommenger et al., 2006; Grundmann et al., 2010; Harrison et al., 2014; Vincze et al., 2014; Couto et al., 2016; Wipf and Perreten, 2016). The CC398 LA-MRSA has also been sporadically reported in cats and dogs, but always as a minor lineage (Haenni et al., 2012; Vincze et al., 2014; Wipf and Perreten, 2016).

Methicillin-resistant *Staphylococcus aureus* epidemiology in horses presents two distinct time phases. Until the early 2000s, a majority of the reported strains in North America and Europe belonged to the clonal complex (CC)8, with a predominance of the CC8-IV USA500 clone, and clustered into the ST254 and ST8 (Weese et al., 2005a; Moodley et al., 2006; Cuny et al., 2008; Walther et al., 2009; van Balen et al., 2014). Most of these CC8 strains likely originated from humans, and subsequently adapted to horses and disseminated in the equine host, independent to the human epidemiology (Weese and van Duijkeren, 2010). After 2004, the emergence and dissemination of a CC398 MRSA clone [Staphylococcal chromosomal cassette *mec* (SCC*mec*) IV, *spa*-type t011] has been reported, from potential direct or indirect livestock origin (Cuny et al., 2008; Van den Eede et al., 2009; van Duijkeren et al., 2010), and this clone is now massively prevalent in horses worldwide. A CC398-IV-t011 MRSA sub-lineage – named clade C – has also been described, which most probably encompasses most of the CC398 MRSA that have already been described in hospitalized horses and veterinarians in contact (Abdelbary et al., 2014). This clade C is presenting a specific single-nucleotide polymorphism (SNP) consisting in a synonymous substitution in the SNP au309-2. It represents nearly 90% of the MRSA isolated from equine wound infections (Vincze et al., 2014) in Germany. A recent study shows it present since 2010 in France (Guerin et al., 2017). An important characteristic of the CC398 isolates is the potential presence of the Immune Evasion Cluster (IEC), composed of combined virulence factors, such as staphylococcal complement inhibitor (*scn*), chemotaxis inhibitory protein (*chp*), staphylokinase (*sak*), and specific staphylococcal enterotoxin genes such as *sea* and *sep* (Hau et al., 2015). This bacteriophage-encoded cluster is a marker of re-adaptation to the human host and enhances its human-to-human transmission capacity.

Methicillin-resistant *Staphylococcus aureus* colonizing or infecting companion animals have become of public health concern since transmissions between animals and humans have been documented many times (Weese et al., 2005b; Walther et al., 2009; van Duijkeren et al., 2010; Bergstrom et al., 2012; Van den Eede et al., 2013). In France, MRSA clones involved in human infections have been reported in specific studies in cats and dogs (Haenni et al., 2012) and recently in horses (Guerin et al., 2017). However, there is still no nationwide comprehensive molecular information on MRSA circulating in cats, dogs, and horses in France, and this was investigated and clarified here over a 5-year period through the French national network for surveillance of resistant bacteria in animals (Resapath^1^). This study was lined up with the 5-year French action plan EcoAntibio set up by the Ministry of Agriculture, Agri-Food and Forestry^2^ in 2011. Its first and recently achieved goal was to reduce animal exposure to antibiotics by 25%. The data presented here are of major importance for risk-assessment studies on MRSA transmission in a One-Health perspective.

## Materials and Methods

### Bacterial Strains

From December 2010 to April 2015, antimicrobial susceptibility test (AST) data of all coagulase-positive staphylococci isolated from horses, cats, and dogs (Supplementary Table S1) were collected through the Resapath network. The Resapath is a network of 78 veterinary laboratories that are transmitting on a voluntary basis all their AST data as well as the strains of interest to the ANSES (French agency for Food, Environmental and Occupational Health Safety). Only clinical isolates were considered in the frame of this study since the Resapath network does not encompass bacteria colonizing animals. All cefoxitin-intermediate or cefoxitin-resistant isolates, as tested by laboratories member of Resapath using the disk diffusion method (see below), were sent to the ANSES laboratory in Lyon for further characterization. After confirmation of the phenotype by disk diffusion, two PCRs were systematically performed: a triplex PCR targeting the 16S rRNA, *mecA* and *nuc* genes to detect the presence of MRSA (Maes et al., 2002), and a simplex PCR using *mecC*-specific primers to detect non-*mecA* MRSA (Garcia-Alvarez et al., 2011). Species identification was then further assessed using the PCR-RFLP designed by Blaiotta et al. (2010) to differentiate *S. aureus* from *S. pseudintermedius*, which is the most frequently encountered coagulase-positive *Staphylococcus* in dogs.

### Susceptibility Testing

Antimicrobial susceptibility test was confirmed by the ANSES laboratory using the disk diffusion method and interpreted according to the guidelines of the Antibiogram Committee of the French Society for Microbiology^3^. *S. aureus* ATCC 25923 was used as quality control. In addition, 16 antibiotics of veterinary and/or human interest were tested: penicillin G, cefoxitin, cefovecine, kanamycin, gentamicin, tobramycin, tetracycline, erythromycin, spiramycin, lincomycin, chloramphenicol, florfenicol, fusidic acid, enrofloxacin, vancomycin, teicoplanin (Mast Diagnostics, Amiens, France).

### Molecular Typing

The presence of the *mecA*/*mecC* gene was systematically confirmed by PCR (Maes et al., 2002; Garcia-Alvarez et al., 2011). Specific detection of the CC398 by PCR was also systematically performed (Stegger et al., 2011). This PCR is based on the detection of a specific *sau1*-*hsdS1* gene (responsible for the restriction modification specificity), which is conserved among the CC398 isolates but differs from other MRSA clones. The *spa*-types were determined and assigned as previously described^4^ (Koreen et al., 2004).

### DNA Microarray

All *mecA*/*mecC*-positive strains were characterized using microarray-based assay (*S. aureus* Genotyping, Identibac – Alere) allowing detection of virulence and resistance genes, SCC*mec* types, and assignment to clones and/or MLST clonal complexes. Protocols, target genes for virulence and resistance, databases, typing information, and interpretation management were available at the following internet address: https://alere-technologies.com/fileadmin/Media/Downloads/op/10620/Manuals/05_16_04_0001_V05_Manual_S.aureus_Genotyping_Kit_2_0.pdf.

## Results

### Epidemiological Data and Antibiotic Resistance of MRSA in Cats, Dogs, and Horses

**FIGURE 1:**
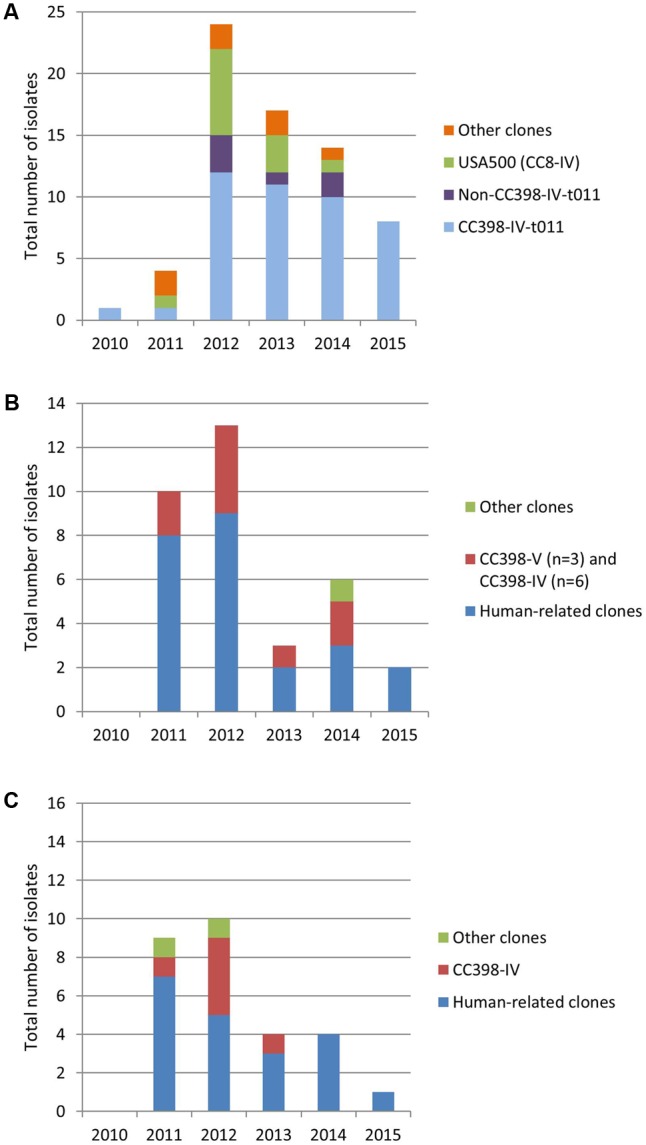
Distribution of clones per year in horses **(A)**, cats **(B)**, and dogs **(C)**.

A total of 130 consecutive non-duplicate clinical MRSA isolates were collected from 43 different French districts: 68 isolates from horses, 34 from cats, and 28 from dogs (Supplementary Table S1). Since 2012, the total number of MRSA collected has significantly decreased in all three animal species (**Figure 1**), despite the fact that the total number of coagulase-positive isolates collected (susceptible and resistant to methicillin) followed either a steady or increasing trend.

None of the MRSA infected animals presented any epidemiological links since they all originated from different cities, departments or regions, and none of the cats or dogs came from a kennel. MRSA were mostly isolated from skin and soft tissue infections (SSTI) [horse, *n* = 32 (32/68, 47.1%); dog, *n* = 11 (11/28, 39.3%); cat, *n* = 9 (9/34, 26.5%)], while MRSA from reproductive tract infections were also highly prevalent in horses (22/68, 32.4%) (Supplementary Table S2). Isolates globally presented high levels of resistance to several antibiotics, particularly to aminoglycosides (50.0% to gentamicin, 70.0% to kanamycin, 67.7% to tobramycin), tetracyclines (60.0%) and enrofloxacin (48.50%) (**Table 1**). Major differences were however observed between the CC398 and human-related isolates, with resistances to tetracyclines, phenicols, and aminoglycosides being more prevalent in CC398 isolates, while resistances to macrolides-lincosamides, fusidic acid, and enrofloxacin were more prevalent in human-related isolates (**Table 1**).

**Table 1:** Resistance to non-beta-lactam antibiotics among MRSA isolated from companion animals.

Antibiotics	Total of MRSA isolates (*n* = 130)		MRSA isolates belonging to human-related clones^a^ (*n* = 46)		MRSA isolates belonging to CC398 clones (*n* = 64)	
	Number of resistant isolates	Percentage of resistant isolates	Number of resistant isolates	Percentage of resistant isolates	Number of resistant isolates	Percentage of resistant isolates
Kanamycin	91	70.0	27	58.7	52	81.3
Tobramycin	88	67.7	30	65.2	54	84.4
Gentamicin	65	50.0	2	4.3	51	79.7
Chloramphenicol	27	20.8	3	6.5	14	21.9
Florfenicol	6	4.6	0	0.0	6	9.4
Tetracycline	78	60.0	5	10.9	61	95.3
Erythromycin	43	33.1	23	50.0	7	10.9
Spiramycin	21	16.2	14	30.4	7	10.9
Lincomycin	23	17.7	14	30.4	8	12.5
Fusidic acid	7	5.4	5	10.9	0	0.0
Enrofloxacin	63	48.5	41	89.1	21	32.8

*^a^Strains considered as human-related MRSA clones are listed in Supplementary Table S2 (underlined strain numbers).*

### Distribution of MRSA Clones in Horses

**FIGURE 2:**
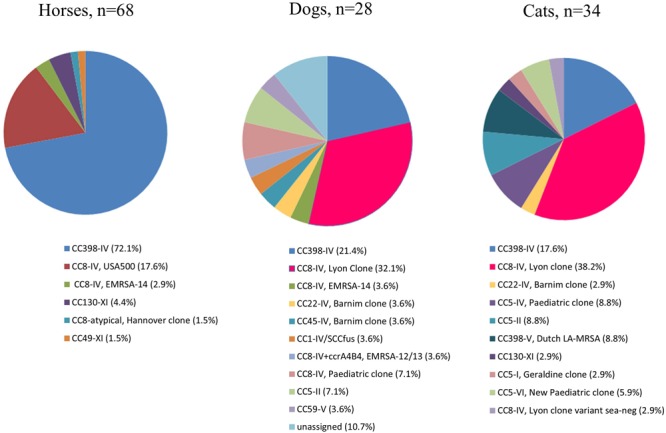
Schematic representation of all clones associated to horses, cats, and dogs. In the dog section the “unassigned” clones corresponded to strains that could not be assigned to a specific clone by DNA array. These three strains corresponded to CC-*spa* types CC30-t012, CC5-t450, and CC8-t2054 (also see Supplementary Table S2). For all strains, associated *spa*-types can be found in Supplementary Table S2.

The most prevalent MRSA clone in horses was the CC398 MRSA clone presenting a SCC*mec* type IV (hereafter named CC398-IV), represented in forty-nine MRSA isolates (49/68, 72.1%) (**Figures 1, 2** and Supplementary Table S2). The *spa*-type t011 was identified in the majority of the isolates (*n* = 42/49, 85.7%), while t899 (*n* = 2), t779 (*n* = 2), t108 (*n* = 1), t1451 (*n* = 1) and one non-typeable isolate were also detected. Conversely to cats and dogs, only two isolates displayed enterotoxin genes [i.e., *sek*/*seq* (*n* = 1) and *sea* (*n* = 1)]. The IEC was present in 12.2% (*n* = 6, including 5 type B and 1 type D) of the 49 CC398 isolates. Tetracycline resistance, which has been strongly associated with CC398 strains from livestock so far (Price et al., 2012), was systematically detected and the *tet*(M) gene identified in 48 of the 49 isolates. The *aacA*-*aphD* gene conferring resistance to gentamicin, kanamycin/amikacin, and tobramycin was also frequently identified (46/49, 93.9%) and correlated with phenotypic resistance (Supplementary Table S2). The other resistance genes were scarce, with the sporadic occurrence of genes conferring resistances to macrolides/lincosamides/streptogramins B [*erm(A), n* = 3], streptogramin A [*vga*(A), *n* = 2], trimethoprim (*dfrS1, n* = 2), fosfomycin (*fosB, n* = 1), chloramphenicol (*cat, n* = 1), tetracyclines [*tet*(K), *n* = 2], or quaternary ammonium compounds (*qacC, n* = 1).

Twelve MRSA (17.6%) isolates clustered with the CC8-IV USA500 clone [*spa*-type t394 (*n* = 8) and t1952 (*n* = 1)]. All isolates presented the *seb, sek*, and *seq* enterotoxin genes, and the *tet*(M), *aacA*/*aphD, cat, dfr*(S1), and *fos*(B) resistance genes were detected in all t394 isolates (Supplementary Table S2). None of them presented the IEC cluster that is classically related to human origin/adaptation.

Among the seven isolates belonging neither to the CC398-IV nor the CC8-IV USA500 clone, three were assigned to the CC8-IV EMRSA-14 (*n* = 2) or to the CC8 Hannover EMRSA (*n* = 1) clones. This CC8 Hannover clone belonged to the ST249-t009 and presented an atypical SCC*mec* element comparable to those described by Monecke et al. (2011). All three strains presented multiple resistance genes, including to tetracyclines (Supplementary Table S2). The last four isolates harbored the *mecC* gene and have already been reported in the frame of another study (Haenni et al., 2015). They belonged to the CC130-XI-t6220, CC130-XI-t1736, CC130-XI-t11050, and CC49-XI-t208 clones (Supplementary Table S2). These four *mecC*-positive isolates were the only equine isolates that were not resistant to tetracyclines and presented no other resistance genes.

### Distribution of MRSA Clones in Cats

Most isolates belong to the prototypic CC8-IV Lyon clone (13/34, 38.2%) or its *sea*-negative variant (*n* = 1). The majority have *spa*-type t008 (*n* = 8) (**Figure 1** and Supplementary Table S2). In addition to these 14 isolates, another 10 isolates belong to human-related clones: CC5-IV Pediatric (*n* = 3) and New Pediatric (*n* = 2) clones, CC5-II (*n* = 3), CC22-IV Barnim (*n* = 1), CC5-I Geraldine (*n* = 1) clones. Among the latter, the IEC (marker of human adaptation) was detected in a vast majority of the isolates (*n* = 17; 17/24, 70.8%). The IEC type A (*sea, sak, scn, chp*) was identified in one isolate, the IEC type B (lacking *sea*) in four isolates, and the IEC type D (lacking *chp*) in 12/13 isolates from the Lyon clone (van Wamel et al., 2006). Among the human-related clones, only one isolate was resistant to tetracyclines without *tet*(M) gene.

On the contrary, the *tet*(M) gene was present in all nine isolates belonging to the CC398 MRSA clone, among which 6 harbored SCC*mec* type IV (CC398-IV; including four isolates with *spa* type t011) and three of the SCC*mec* type V (CC398-V). The IEC cluster was only found in three isolates [type B (*n* = 1) or type D (*n* = 2)]. All CC398-V clones presented at least the *tet*(M) and *fexA* resistance genes, whereas the CC398-IV clone carried the *tet*(M) and *aacA*-*aphD* genes. The co-occurrence of the *tet*(M) and *tet*(K) genes was identified in two *spa*-type t034 CC398-V. This association, recently shown to increase the fitness of CC398-V strains in the presence of tetracyclines (Larsen et al., 2016), was also identified here in a t1451 CC398-IV isolate originating from a horse (Supplementary Table S2). Finally, one isolate, belonging to the CC130-XI t843 clone, harbored the *mecC* gene with no associated resistance or enterotoxin genes.

### Distribution of MRSA Clones in Dogs

As for cats, nine isolates belonged to the CC8-IV Lyon clone (32.1%) or to other sporadically occurring human-related clones (*n* = 10) (**Figure 1** and Supplementary Table S2). A total of 16 out of the 28 isolates displayed the IEC cluster [type B, *n* = 6; type D, *n* = 10 (including the nine Lyon clone isolates)]. Only one isolate did not present any associated enterotoxin gene. Resistance phenotypes to macrolides (10/28, 35.7%), aminoglycosides (15/28, 53.6%), and fosfomycin (18/28, 64.3%) were frequently encountered, whereas only one isolate was resistant to tetracyclines in the absence of the *tet*(M) gene. The CC398-IV clone was identified in six tetracycline-resistant isolates (21.4%), among which two presented the IEC cluster (type B and type D). The last three isolates belonged to the CC-*spa* types CC30-t012, CC5-t450, and CC8-t2054 but could not be assigned to any specific clone by the DNA array. Finally, no *mecC* gene was detected in dogs.

## Discussion

This study describes the largest collection of MRSA isolates ever collected in France from companion animals (horses, cats, and dogs). It can be compared with the MRSA collection from these three animal species published by Vincze et al. (2014) in Germany, and also to the data very recently published in a specific study focused on MRSA in horses by Guerin et al. (2017) in France.

In our study, only one MRSA was isolated from horses in 2010, likely due to a poor representation of horses, cats and dogs in the Resapath network at the time (the total number of coagulase-positive staphylococci reported in 2010 was respectively 5 in horses, 11 in cats, 88 in dogs). Since 2011, this gap has been progressively filled in and the obvious decrease in the total number of MRSA collected over years (**Figure 1**) likely reflects a true decrease in the prevalence of MRSA in horses, cats, and dogs in France. A major reason for this decrease may well be the recent global reduction in the use of antibiotics in the veterinary field in France, in line with the 5-year national action plan EcoAntibio of the Ministry of Agriculture, Agri-Food and Forestry. Indeed, a decrease (from 19.4% to 28.1%) was observed in the global exposure to antibiotics of respectively cats/dogs and horses^5^. Such a valuable trend will be thoroughly monitored in the near future.

### Success in Horses of the CC398-IV MRSA Clone Belonging to the *spa*-Type t011

The molecular characterization of the horse collected isolates highlights that MRSA identified in these animals are independent to the human MRSA epidemiology. Two major animal-associated clones were identified in France: the CC8-IV USA500 and the CC398-IV-t011 clones. The CC8-IV USA500, which is the predominant clone infecting horses in North America, also recently described as an environmental contaminant in an equine veterinary center (Weese et al., 2005a; van Balen et al., 2014), was detected in France between 2011 and 2013, with a last occurrence in early 2014. This suggests that this clone, uncommon in the human population in France and worldwide, is prone to colonize and/or infect horses worldwide. Yet CC8-IV USA500 seems to have been totally replaced by the horse-specific CC398-IV belonging to the *spa*-type t011 in France (**Figure 1**), confirming its international spread and high fitness/adaptation to horses. Between 2013 and 2015, only 7/39 (17.9%) isolates did not belong to the CC398 clone. Of note, four out of these seven isolates displayed the *mecC* gene. Whether horses may be prone to disseminate *mecC* genes or not remains questionable. Both in terms of management by veterinarians and with regards to the global burden antimicrobial resistance, the replacement of CC8-IV USA500 by the CC398-IV-t011 clone may be considered good news since CC398 were co-resistant to aminoglycosides and tetracyclines only, whereas USA500 additionally displayed resistances to macrolides-lincosamides, chloramphenicol, sulfonamides, and fosfomycin. Even though these antibiotic molecules are not all licensed for horse treating, any decrease in antibiotic resistance burden is to be noticed since it may also decrease the risk of co-selection of resistant isolates in other animal species or in humans. This is of course only on a resistance perspective, and the differences in virulence capacities (such as biofilm formation or the presence of adhesion factors) will also have to be considered.

Considering the nosocomial and zoonotic potential of MRSA isolated from horses, equine veterinarians should pay specific attention to both antibiotic treatments and hygiene measures, to limit MRSA selection and transmission. The rate of MRSA carriage in healthy horses, as well as the potential human-to-animal or animal-to-human transmission in both veterinary clinics and in equestrian centers was studied on certain occasions and certainly deserve further investigation (van Duijkeren et al., 2010; Cuny et al., 2016; Koop, 2016).

### Human-Related MRSA Clones in Cats and Dogs

Our data also underline that MRSA isolated from cats and dogs mirror the epidemiology of hospital-acquired human MRSA (Loeffler and Lloyd, 2010). The CC8-IV Lyon clone was the most frequently identified MRSA clone in both animal species, followed by the occurrence of numerous sporadic lineages. This is in accordance with a similar study previously conducted in France on a smaller number of isolates (Haenni et al., 2012). The ST22 Barnim clone is again rare in France despite its high prevalence in animals in surrounding countries (Strommenger et al., 2006; Grundmann et al., 2010; Harrison et al., 2014; Vincze et al., 2014; Couto et al., 2016; Wipf and Perreten, 2016). Its rare occurrence is most likely related to its low prevalence in French hospitals and in the community. The CC398 clone was detected in 21.4% of all dogs’ and 26.5% of all cats’ isolates, which is more frequent that what has been reported in Germany and much more than what has been recently observed in France (2/23, 8.7%) or in other European countries (Haenni et al., 2012; Vincze et al., 2014; Wipf and Perreten, 2016). Since no links could be inferred between animals sampled and pig farming or contact with livestock, the hypothesis of a progressive replacement of existing human MRSA clones in cats and dogs by the CC398 cannot be excluded, as observed in horses,. Data collected are too limited to confirm such an hypothesis and this will deserve to be followed-up, yet CC398 clones disseminated among humans have recently been exemplified in Denmark, not all to be attributed to contact with livestock (Larsen et al., 2015). Interestingly, all CC398 isolates presented the *tet*(M) gene, whereas all non-CC398 were devoid of the gene. Other *tet* genes being rare (Supplementary Table S2), tetracycline susceptibility in MRSA from cats and dogs may be used as a marker for human-related isolates, mirroring the fact that tetracycline-resistance [or the *tet*(M) presence] can be used as a marker for MRSA CC398 in humans living in high pig-farming areas (Lozano et al., 2012).

## Conclusion

We show here a divergent epidemiology between cats/dogs and horses, with the first two species being largely colonized by human-related isolates mirroring the human MRSA epidemiology. These strains produce enterotoxins more frequently and display a higher prevalence of the IEC cluster than the animal-associated MRSA isolates colonizing horses. Considering the proximity of humans with their pets and the possibility of human re-infection, prevalence of MRSA in cats and dogs is undoubtedly a public health issue that deserves to be monitored. Conversely, horses are mostly infected by animal-associated isolates that differ from the traditional ST398 LA-MRSA with the particular prevalence of the CC398-IV MRSA clone belonging to the *spa*-type t011. Cats and dogs are also infected by CC398 MRSA clones, and special attention will have to be paid to the IEC-positive CC398 MRSA isolates, whose prevalence was substantial in our study (11/64, 17.2%). This rate is particularly high compared to the one detected in LA-MRSA from in MRSA from horses in Germany (∼10%) (Cuny et al., 2015). Of note, no IEC-positive CC398 MRSA had been detected from pigs’ isolates in the same study. Re-adaptation to a human host, due to closer contacts between companion animals and humans compared to pigs and farmers, may favor virulence and animal-independent dissemination of such MRSA isolates in humans (Stegger et al., 2013). Taken together, this set of data promote a regular surveillance of MRSA in companion animals in the coming years to assess whether the CC398 would replace human-related MRSA clones in cats and dogs and whether those clones will be able to re-adapt to the human host.

## Author Contributions

MH, J-YM, MB, and FL designed the experiments. PC, VM, CD-C, and MB did the experiments. MH and FL analyzed the data. MH and FL drafted the manuscript. J-YM and MB actively contributed to the manuscript’s writing. All authors approved the final version of this manuscript.

## Conflict of Interest Statement

The authors declare that the research was conducted in the absence of any commercial or financial relationships that could be construed as a potential conflict of interest.

## Footnotes

1 https://www.resapath.anses.fr/

2 http://agriculture.gouv.fr/ecoantibio

3 www.sfm-microbiologie.org/

4 http://www.spaserver.ridom.de/

5 https://www.anses.fr/en/content/monitoring-sales-veterinary-antimicrobials
