# Supplementary material for: Molecular Epidemiology of Methicillin-Resistant Staphylococcus aureus in Horses, Cats, and Dogs Over a 5-Year Period in France
Source: Front Microbiol. 2017 Dec 13;8:2493. doi: 10.3389/fmicb.2017.02493 (PMC5733339; doi:10.3389/fmicb.2017.02493)
Supplement: Supplementary file 1 [file Table_1.PDF]

Table S1. Total number of coagulase-positive staphylococci screened and of MRSA confirmed

	Year						
	2010	2011	2012	2013	2014	2015	total
Number of coagulase-positive staphylococci screened							
Horses	5	62	255	313	337	109	1081
Cats	11	161	173	269	255	53	922
Dogs	88	1431	1784	2134	2046	512	7995
Number of MRSA confirmed[a]							
Horses	1	4	24	17	14	8	68
Cats	0	10	13	3	6	2	34
Dogs	0	9	10	4	4	1	28

[a] the MRSA prevalence cannot be inferred since coagulase-positive staphylococci encompass not only *S. aureus* but also other species such as *S. pseudintermedius*.
