# Supplementary material for: Molecular Epidemiology of Methicillin-Resistant Staphylococcus aureus in Horses, Cats, and Dogs Over a 5-Year Period in France
Source: Front Microbiol. 2017 Dec 13;8:2493. doi: 10.3389/fmicb.2017.02493 (PMC5733339; doi:10.3389/fmicb.2017.02493)
Supplement: Supplementary file 2 [file Table_2.pdf]

Table S2. Molecular and epidemiological characteristics of the 130 MRSA isolates

Isolate	Year	Animal host	Pathology	District	Clone	IEC[d]	Resistance genes	Enterotoxins genes	*Spa*-type
26023	2010	Horse	Reproductive tract	33	CC398-IV	no	*aacA-aphD*, *tet*(M)	none	t011
31749[a]	2011	Horse	Reproductive tract	61	CC8-IV, EMRSA-14	no	*aacA-aphD*, *tet*(M), *dfrS1*, *fosB*	none	t064
32084	2011	Horse	Reproductive tract	50	CC8-IV, USA500	no	*erm*(C), *aacA-aphD*, *tet*(M), *dfrS1*, *fosB*	*seb*/*sek*/*seq*	t1952
32085	2011	Horse	Reproductive tract	50	CC8-IV, EMRSA-14	no	*erm*(C), *tet*(M), *dfrS1*, *fosB*	none	t1952
32090	2011	Horse	Reproductive tract	50	CC398-IV	no	*aacA-aphD*, *tet*(M)	none	t011
32141	2012	Horse	SSTI[b]	14	CC398-IV	no	*aacA-aphD*, *tet*(M)	none	t011
32145	2012	Horse	SSTI	78	CC398-IV	no	*aacA-aphD*, *tet*(M)	*sek*/*seq*	t011
32147	2012	Horse	Reproductive tract	74	CC398-IV	no	*aacA-aphD*, *tet*(M)	none	t779
32150	2012	Horse	SSTI	76	CC398-IV	no	*aacA-aphD*, *tet*(M)	none	t011
32151	2012	Horse	Reproductive tract	50	CC398-IV	no	*aacA-aphD*, *tet*(M), *cat*	none	t011
32166	2012	Horse	Reproductive tract	50	CC398-IV	no	*aacA-aphD*, *tet*(M)	none	t779
32167	2012	Horse	SSTI	34	CC398-IV	type B	*vga*(A), *tet*(M)	none	t899
32347	2012	Horse	Reproductive tract	33	CC398-IV	no	*aacA-aphD*, *tet*(M), *cat*	none	t011
32716	2012	Horse	SSTI	49	CC398-IV	type D	*erm*(A), *aadD*, *fosB*	*sea*	t011
32718	2012	Horse	Bone infection	92	CC8-IV, USA500	no	*erm*(C), *aacA-aphD*, *tet*(M), *cat*, *dfrS1*, *fosB*	*seb*/*sek*/*seq*	t394
32719	2012	Horse	SSTI	92	CC8-IV, USA500	no	*erm*(C), *aacA-aphD*, *tet*(M), *cat*, *dfrS1*, *fosB*	*seb*/*sek*/*seq*	t394
32720	2012	Horse	SSTI	92	CC8-IV, USA500	no	*erm*(C), *aacA-aphD*, *tet*(M), *cat*, *dfrS1*, *fosB*	*seb*/*sek*/*seq*	t394
32721	2012	Horse	SSTI	91	CC8-IV, USA500	no	*erm*(C), *aacA-aphD*, *tet*(M), *cat*, *dfrS1*, *fosB*	*seb*/*sek*/*seq*	t394
32722	2012	Horse	Reproductive tract	10	CC398-IV	no	*aacA-aphD*, *tet*(M)	none	t011
32723	2012	Horse	Respiratory tract infection	1	***CC130-XI***[c]	no	none	none	t6220
32777	2012	Horse	SSTI	10	CC398-IV	no	*aacA-aphD*, *tet*(M)	none	t011
32779	2012	Horse	SSTI	89	CC8-IV, USA500	no	*erm*(C), *aacA-aphD*, *tet*(M), *cat*, *dfrS1*, *fosB*	*seb*/*sek*/*seq*	t394
32780	2012	Horse	SSTI	92	CC398-IV	no	*aacA-aphD*, *tet*(M)	none	t011
32781	2012	Horse	Respiratory tract infection	67	CC8-atypical SCC*mec*, Hannover clone	no	*aacA-aphD*, *aph3*, *sat*, *tet*(M), *fosB*	*seb*/*sek*/*seq*	t009
32782	2012	Horse	SSTI	90	CC398-IV	no	*aacA-aphD*, *tet*(M)	none	t011
32960	2012	Horse	Reproductive tract	1	CC398-IV	type B	*aacA-aphD*, *tet*(M)	none	t011
32970	2013	Horse	SSTI	92	CC8-IV, USA500	no	*erm*(C), *aacA-aphD*, *tet*(M), *cat*, *dfrS1*, *fosB*	*seb*/*sek*/*seq*	t394
32971	2012	Horse	SSTI	44	CC398-IV	no	*aacA-aphD*, *tet*(M)	none	t011

32973	2013	Horse	SSTI	14	CC398-IV	no	*aacA-aphD*, *tet*(M)	none	t011
32975	2012	Horse	SSTI	78	CC8-IV, USA500	no	*erm*(C), *aacA-aphD*, *tet*(M), *cat*, *dfrS1*, *fosB*	*seb*/*sek*/*seq*	t394
32976	2013	Horse	SSTI	92	CC398-IV	no	*aacA-aphD*, *tet*(M)	none	t011
32977	2012	Horse	SSTI	92	CC8-IV, USA500	no	*erm*(C), *aacA-aphD*, *tet*(M), *cat*, *dfrS1*, *fosB*	*seb*/*sek*/*seq*	t394
33329	2013	Horse	SSTI	92	CC8-IV, USA500	no	*erm*(C), *aacA-aphD*, *tet*(M), *cat*, *dfrS1*, *fosB*	*seb*/*sek*/*seq*	t394
33330	2013	Horse	Respiratory tract infection	78	CC8-IV, USA500	no	*erm*(C), *aacA-aphD*, *tet*(M), *cat*, *dfrS1*, *fosB*	*seb*/*sek*/*seq*	t394
33584	2013	Horse	NP	12	CC398-IV	type B	*erm*(B), *vga*(A), *tet*(M), *dfrS1*	none	t899
33589	2013	Horse	Reproductive tract	50	CC398-IV	no	*aacA-aphD*, *tet*(M)	none	t011
33594	2013	Horse	Bone infection	61	CC398-IV	no	*aacA-aphD*, *tet*(M), *cat*	none	t011
33709	2013	Horse	Reproductive tract	14	CC398-IV	no	*aacA-aphD*, *tet*(M)	none	t108
33710	2013	Horse	Abdominal surgery	77	CC398-IV	no	*aacA-aphD*, *tet*(M)	none	t011
33868	2013	Horse	Bone infection	61	CC398-IV	no	*aacA-aphD*, *tet*(M)	none	t011
33871	2013	Horse	Reproductive tract	61	CC398-IV	no	*aacA-aphD*, *tet*(M)	none	t011
33996	2013	Horse	SSTI	95	***CC49-XI***	no	none	*sec*/*sel*	t208
35412	2013	Horse	SSTI	76	***CC130-XI***	no	none	none	t1736
35846	2013	Horse	NP	74	CC398-IV	type B	*aacA-aphD*, *tet*(M)	none	t011
35847	2013	Horse	NP	16	CC398-IV	no	*aacA-aphD*, *tet*(M), *cat*	none	t011
35851	2014	Horse	SSTI	92	CC8-IV, USA500	no	*erm*(C), *aacA-aphD*, *tet*(M), *cat*, *dfrS1*, *fosB*	*seb*/*sek*/*seq*	t394
35855	2014	Horse	SSTI	47	CC398-IV	no	*aacA-aphD*, *tet*(M)	none	txAJ
38399	2014	Horse	Reproductive tract	31	CC398-IV	no	*aacA-aphD*, *tet*(M)	none	t011
38400	2014	Horse	Abdominal surgery	77	CC398-IV	no	*aacA-aphD*, *tet*(M)	none	t011
38414	2014	Horse	Reproductive tract	61	CC398-IV	no	*aacA-aphD*, *tet*(M), *cat*	none	t011
38423	2014	Horse	SSTI	14	CC398-IV	no	*aacA-aphD*, *tet*(M)	none	t011
38425	2014	Horse	Bone infection	61	CC398-IV	no	*aacA-aphD*, *tet*(M), *cat*	none	t011
38494	2014	Horse	Reproductive tract	14	CC398-IV	no	*aacA-aphD*, *tet*(M)	none	t011
38497	2013	Horse	NP	NP	CC398-IV	no	*aacA-aphD*, *tet*(M)	none	t011
38570	2014	Horse	SSTI	45	***CC130-XI***	no	none	none	t11050
38571	2014	Horse	Abdominal surgery	37	CC398-IV	no	*aacA-aphD*, *tet*(M)	none	t011
38713	2014	Horse	SSTI	77	CC398-IV	no	*aacA-aphD*, *tet*(M)	none	t011
38719	2014	Horse	SSTI	14	CC398-IV	no	*aacA-aphD*, tet(M), *dfrS1*, *qacC*	none	t011
38720	2014	Horse	SSTI	33	CC398-IV	no	*aacA-aphD*, *tet*(K), *tet*(M)	none	t1451
39653	2015	Horse	Reproductive tract	14	CC398-IV	no	*aacA-aphD*, *tet*(M)	none	t011
39655	2015	Horse	SSTI	77	CC398-IV	no	*aacA-aphD*, *tet*(M), *cat*	none	t011
39680	2015	Horse	Reproductive tract	19	CC398-IV	no	*aacA-aphD*, *tet*(M)	none	t011
39681	2014	Horse	SSTI	75	CC398-IV	no	*aacA-aphD*, *tet*(M)	none	t011
40087	2015	Horse	SSTI	78	CC398-IV	no	*erm*(C), *aacA-aphD*, *tet*(M)	none	t011

40088	2015	Horse	SSTI	94	CC398-IV	type B	*aacA-aphD*, *tet*(M)	none	t011
40089	2015	Horse	Reproductive tract	14	CC398-IV	no	*aacA-aphD*, *tet*(M)	none	t011
40090	2015	Horse	Reproductive tract	14	CC398-IV	no	*aacA-aphD*, *tet*(M)	none	t011
40096	2015	Horse	Reproductive tract	61	CC398-IV	no	*aacA-aphD*, *tet*(M)	none	t011
25175	2011	Cat	SSTI	6	CC5-IV, Paediatric clone	no	*erm*(C), *aadD*, *fosB*	*seg/sei/sem/sen/seo/seu*	t002
26451	2011	Cat	Digestive tract infection	76	CC8-IV, Lyon Clone	type D	*erm*(A), *aadD*, *fosB*	*sea*	t068
26693	2011	Cat	Respiratory tract infection	94	CC5-VI, New Paediatric Clone	no	*fosB*	*sed/seg/sei/sej/sem/sen/ seo/ser/seu*	t777
26695	2011	Cat	NP	94	CC8-IV, Lyon Clone	type D	*erm*(A), *aadD*, *fosB*	*sea*	t574
26741	2012	Cat	NP	94	CC8-IV, Lyon Clone	type D	*erm*(A), *aadD*, *fosB*	*sea*	t574
27101	2011	Cat	Respiratory tract infection	94	CC5-VI, New Paediatric Clone	no	*fosB*	*sed/seg/sei/sej/sem/sen/ seo/ser/seu*	t777
27272	2011	Cat	NP	94	CC398-IV	no	*aacA-aphD*, *tet*(M)	none	t011
27744	2011	Cat	NP	94	CC22-IV, Barnim clone	type B	none	*seg/sei/sem/sen/seo/seu*	t032
31929	2011	Cat	Bone infection	54	CC5-II	type A	*erm*(A), *fosB*	*sea/sed/seg/sei/sej/sem/sen/ seo/ser/seu*	t003
32305	2012	Cat	Urinary tract	6	CC8-IV, Lyon Clone	type D	*aadD*, *fosB*	*sea/sed/sej/ser*	t008
32610	2012	Cat	Urinary tract	94	CC8-IV, Lyon Clone	type D	*erm*(A), *aadD*, *fosB*	*sea*	t008
32615	2012	Cat	SSTI	56	CC398-V, Dutch LA-MRSA	no	*erm*(C), *tet*(K), *tet*(M), *fexA*	none	t034
32616	2012	Cat	Urinary tract	56	CC398-V, Dutch LA-MRSA	no	*erm*(C), *tet*(K), *tet*(M), *fexA*	none	t034
32654	2012	Cat	SSTI	92	CC398-IV	no	*aacA-aphD*, *tet*(M)	none	t011
32664	2012	Cat	SSTI	94	CC8-IV, Lyon Clone	no	*erm*(A), *aadD*, *fosB*	*sea*	t008
32665	2012	Cat	Urinary tract	56	CC398-V, Dutch LA-MRSA	no	*tet*(M), *fexA*	none	t034
32691	2011	Cat	SSTI	77	CC398-IV	type D	*aacA-aphD*, *tet*(M)	none	t011
32698	2011	Cat	NP	92	CC8-IV, Lyon Clone variant sea-neg	no	*aadD*	*sed/sej/ser*	t622
32775	2012	Cat	Urinary tract	14	CC5-II	no	*erm*(A), *aadD*	*sed/seg/sei/sej/sem/sen/ seo/ser/seu*	t003
32810	2012	Cat	Urinary tract	45	CC8-IV, Lyon Clone	type D	*erm*(A), *aadD*	*sea*	t008
32811	2012	Cat	SSTI	57	CC5-I, Geraldine clone	type B	*aadD*	*sec/sed/seg/sei/sej/sel/sem/ sen/seo/ser/seu*	t002
32814	2012	Cat	SSTI	94	CC8-IV, Lyon Clone	type D	*erm*(A), *aadD*	*sea*	t008
32820	2012	Cat	Respiratory tract infection	92	CC5-IV, Paediatric clone	no	none	*sed/seg/sei/sej/sem/sen/ seo/ser/seu*	t777
33648	2013	Cat	Urinary tract	62	CC5-II	type B	*erm*(A), *aadD*, *fosB*	*sed/seg/sei/sej/sem/sen/ seo/sep(/sea) /ser/seu,*	t003

33704	2013	Cat	SSTI	26	CC398-IV	type D	*aacA-aphD*, *tet*(M)	none	t011
34007	2013	Cat	Respiratory tract infection	92	CC8-IV, Lyon Clone	type D	none	*sea/sed/sej/ser*	t008
35964	2014	Cat	SSTI	92	***CC130-XI***	no	none	none	t843
38508	2014	Cat	NP	NP	CC398-IV	no	*dfrS1*, *tet*(M), *fexA*	none	t2922
38705	2014	Cat	NP	75	CC8-IV, Lyon Clone	type D	*erm*(A), *aadD*, *fosB*, *qacA*	*sea*	t3291
39614	2014	Cat	Urinary tract	93	CC8-IV, Lyon Clone	type D	*erm*(A), *aadD*, *fosB*	*sea*	t008
39665	2015	Cat	NP	59	CC8-IV, Lyon Clone	type D	*fosB*	*sea*	t008
39667	2015	Cat	NP	44	CC5-IV, Paediatric clone	type B	*aadD*, *fosB*	*sed/seg/sei/sej/sem/sen/seo/ser/seu*	t067
39669	2014	Cat	Urinary tract	92	CC8-IV, Lyon Clone	type D	*aadD*, *cat*, *fosB*	*sea*	t622
39819	2014	Cat	NP	38	CC398-IV	type B	*vga*(A), *dfrS1*, *tet*(M)	none	t15493
26982	2011	Dog	SSTI	54	CC8-IV, Lyon Clone	type D	*erm*(C), *aadD*, *fosB*	*sea/sed/sej/ser*	t008
27095	2011	Dog	SSTI	94	CC8-IV, EMRSA-14	no	*fosB*, tetEfflux	none	t121
27134	2011	Dog	NP	94	CC8-IV, Lyon Clone	type D	*erm*(A), *aadD*, *fosB*	*sea*	t008
27139	2011	Dog	SSTI	94	CC8-IV, Lyon Clone	type D	*erm*(A), *aadD*, *tet*(K), *fosB*	*sea*	t008
27262	2011	Dog	NP	12	CC398-IV	no	*aadD*, *dfrA*, *tet*(M), *fexA*, *fosB*, tetEfflux	none	t2922
27267	2011	Dog	NP	94	Unassigned (CC8)	type D	*erm*(A), *fosB*, tetEfflux	*sea*	t2054
27741	2011	Dog	SSTI	94	CC8-IV, Lyon Clone	type D	*vga*(A), *fosB*	*sea/sed/sej/ser*	t211
32078	2011	Dog	SSTI	30	CC8-IV Paediatric clone	type B	*aadD*, *fosB*	*seg/sei/sem/sen/seo/seu*	t002
32310	2012	Dog	NP	6	CC8-IV, Lyon Clone	type D	*aadD*, *fosB*	*sea/sed/sej/ser*	t1343
32313	2012	Dog	SSTI	6	CC8-IV+ccrA4B4, EMRSA-12/13	type D	*fosB*	*sea/sed/sej/ser*	t008
32326	2012	Dog	SSTI	30	Unassigned (CC30-)	type D	none	*seg/sei/sem/sen/seo/seu*	t012
32643	2012	Dog	Respiratory tract infection	66	CC8-IV, Paediatric clone	type B	*fusC*, *fosB*	*seg/sei/sem/sen/seo/seu*	t311
32700	2011	Dog	NP	94	CC8-IV, Lyon Clone	type D	*erm*(A), *aadD*, *fosB*	*sea/sed/sej/ser*	t008
32706	2012	Dog	NP	75	CC398-IV	no	*aacA-aphD*, *tet*(M)	none	t008
32732	2012	Dog	SSTI	4	CC398-IV	type B	*vga*(A), *tet*(M)	none	t899
32784	2012	Dog	Abdominal surgery	75	CC45-IV, Barnim clone	type B	*erm*(C)	*sec/seg/sei/sel/sem/sen/seo/seu*	t025
32978	2012	Dog	Otitis	91	CC398-IV	no	*aacA-aphD*, *tet*(M)	none	t011
32983	2012	Dog	Respiratory tract infection	13	CC59-V	type B	*msr*(A), *farI*, *mupA*, *qacC*	*seb/sek/seq*	t316
33012	2012	Dog	NP	92	CC398-IV	type D	*aacA-aphD*, *tet*(M)	none	t011
33635	2013	Dog	SSTI	57	CC5-II	no	*erm*(A), *fosB*	*sed/seg/sei/sej/sem/sen/seo/ser/seu*	t003
33658	2013	Dog	NP	94	CC8-IV, Lyon Clone	type D	*erm*(A), *aadD*, *fosB*, *qacC*	*sea*	t460
33729	2013	Dog	Reproductive tract	16	CC398-IV	no	*aadD*, *dfrS1*, *tet*(M), *fexA*	none	t2922
33875	2013	Dog	Bone infection	14	CC5-II	type B	*erm*(A), *aadD*, *fosB*	*seg/sei/sem/sen/seo/seu*	t586
38710	2014	Dog	SSTI	42	CC1-IV/SCCfus	no	*fusC*	*seh*	t127

39617	2014	Dog	Reproductive tract	92	CC8-IV, Lyon Clone	type D	*aadD, fosB*	*sea/sed/sej/ser*	t622
39643	2014	Dog	NP	92	CC8-IV, Lyon Clone	type D	*aadD, cat, fosB*	*sea/sed/sej/ser*	t622
39659	2014	Dog	Otitis	6	CC22-IV, Barnim clone	type B	none	*seg/sei/sem/sen/seo/seu*	t15634
40091	2015	Dog	SSTI	85	Unassigned (CC5)	type B	*fusC, fosB*	*sec/sed/seg/sei/sej/sel/ sem/sen/seo/ser/seu*	t450

[a] underlined are all strains considered as belonging to human-related clones
[b] SSTI: skin and soft tissue infection
[c] In bold and italics are the *mecC*-positive isolates
[d] IEC: Immune Evasion Cluster
